# Monensin inhibits mast cell mediated airway contractions in human and guinea pig asthma models

**DOI:** 10.1038/s41598-022-23486-1

**Published:** 2022-11-07

**Authors:** Jielu Liu, Mu Nie, Caijuan Dong, Jesper Säfholm, Gunnar Pejler, Gunnar Nilsson, Mikael Adner

**Affiliations:** 1 Experimental Asthma and Allergy Research Unit, Institute of Environmental Medicine (IMM), Biomedicum, Karolinska Institutet, Solnavägen 9, 17165 Stockholm, Sweden; 2 Department of Medical Biochemistry and Microbiology, Uppsala University, Uppsala, Sweden; 3 Division of Immunology and Allergy, Department of Medicine, Karolinska Institutet and Karolinska University Hospital, Stockholm, Sweden; 4 Department of Medical Sciences, Uppsala University, Uppsala, Sweden

**Keywords:** Mechanisms of disease, Medical research, Experimental models of disease, Translational research

## Abstract

Asthma is a common respiratory disease associated with airway hyperresponsiveness (AHR), airway inflammation and mast cell (MC) accumulation in the lung. Monensin, an ionophoric antibiotic, has been shown to induce apoptosis of human MCs. The aim of this study was to define the effect of monensin on MC responses, *e.g.,* antigen induced bronchoconstriction, and on asthmatic features in models of allergic asthma. Tracheal segments from house dust mite (HDM) extract sensitized guinea pigs were isolated and exposed to monensin, followed by histological staining to quantify MCs. Both guinea pig tracheal and human bronchi were used for pharmacological studies in tissue bath systems to investigate the monensin effect on tissue viability and antigen induced bronchoconstriction. Further, an HDM-induced guinea pig asthma model was utilized to investigate the effect of monensin on AHR and airway inflammation. Monensin decreased MC number, caused MC death, and blocked the HDM or anti-IgE induced bronchoconstriction in guinea pig and human airways. In the guinea pig asthma model, HDM-induced AHR, airway inflammation and MC hyperplasia could be inhibited by repeated administration of monensin. This study indicates that monensin is an effective tool to reduce MC number and MCs are crucial for the development of asthma-like features.

## Introduction

Asthma is a chronic inflammatory disease characterized by airway hyperresponsiveness (AHR)^1^. Thus, asthmatic individuals commonly suffer from recurrent respiratory symptoms, such as wheezing, chest tightness, dyspnea and cough^2^. Mast cells (MCs) are important regulators involving in homeostasis, inflammation initiation and danger prevention^3^. In addition, MCs are participating in diseases. For instance, allergen-induced bronchoconstrictions are mainly due to activation of mast cells (MCs), which are increased in the lungs of asthmatic individuals^4^. When activated, MCs release both preformed and de novo synthesized mediators, such as histamine, prostaglandins (PGs), leukotrienes, cytokines and growth factors^5^. Airway smooth muscle constriction induced by MC degranulation is primarily mediated by activation of the histamine H_1_ receptor, the cysteinyl leukotriene 1 (CysLT_1_) receptor and the thromboxane (TX) receptor (TP)^6–10^. The latter can be activated by different prostanoids, such as PGD_2_, PGE_2_ and PGF_2α_ in addition to TXA_2_^11^. However, the role of MCs in developing AHR of asthma is still ambiguous.

To explore the role of MCs in asthma, there are several potential strategies, such as to prevent MC activation or degranulation, to block MC-secreted mediators or their corresponding receptors, to interfere with MC survival or induce MC apoptosis^12^. However, several of those strategies have demonstrated limited effects. For instance, sodium cromoglycate, a MC stabilizer, showed limited efficacy in inhibiting MC mediator release^13^. Furthermore, even though antagonism of histamine H_1_, CysLT_1_ or TP receptors alone have showed effects on allergen or exercise-induced bronchoconstriction in asthma patients, the combination antagonism is indicated to be even more beneficial^14,15^. This might be due to the complex repertoire of mediators secreted by MCs, where targeting a single mediator pathway may not be sufficient. An alternative strategy, which is gaining increased attention is to selectively target MCs in a specific tissue^12^. In a previous study, we screened 1, 200 off-patented drugs and found monensin, a broadly used ionophoric antibiotic in veterinarian medicine, could selectively induce apoptosis of human and mouse MCs by exploiting the acidic contents of the MC secretory granules^16^. How treatment with monensin affects MC-related actions in tissues and in vivo needs to be further investigated.

The aim of this study was to define the effect of monensin on MC responses, such as antigen induced bronchoconstriction, and on asthmatic features in models of allergic asthma. For this purpose, we performed investigations on how monensin treatment affects the contractile responses in isolated guinea pig trachea and human bronchi which were stimulated with antigen^11,17^ and the airway responsiveness in vivo in a guinea pig asthma model using house dust mite extract (HDM) as allergen^18^. The reason why guinea pig models were used is that guinea pigs have several similarities to humans regarding airway anatomy, physiology, and pharmacology, especially MC location and responses^19^.

## Methods

### Guinea pig sensitization and tissue preparation

Female Dunkin-Hartley guinea pigs (Envigo, Horst, The Netherlands) were housed in the animal facility (KM-F (Astrid Fagræus laboratory), Karolinska Institutet, Solna, Sweden) with free access to food and water. Animals weighing 350–400 g, were sensitized to HDM by a single intraperitoneal (*i.p.*) injection of HDM extract (Lot#369,446, Greer Laboratories, Lenoir, NC, USA) with adjuvant (100 µg/100 mg HDM protein/aluminum hydroxide). Two weeks after sensitization, guinea pigs were euthanized by CO_2_, for carefully isolation of tracheas which were cut into intact rings in cold Krebs–Henseleit buffer. The tracheal segments were cultured in 1 mL Dulbecco modified Eagle medium (Gibco, Auckland, New Zealand) with penicillin (100 IU/mL) and streptomycin (100 mg/mL; Gibco) in a humidified incubator at 37 °C with carbogen (95% O_2_ and 5% CO_2_) overnight under sterile conditions. Monensin (1 or 10 µM) or vehicle (1% ethanol) was added the next day and incubated for another 24 h or 72 h with daily change of medium and addition of new compounds. After culturing, segments were mounted in tissue baths (EMKA, Paris, France) for functional studies.

### Human bronchi isolation and culturing

With the approvement from Stockholm ethics committee (2018/1819–31/1) and consent from patients that underwent lobectomy, healthy human lung tissues were collected and dissected in ice-cold Krebs buffer. Airways with a diameter between 0.5 and 2 mm were isolated, cut into sections and cultured in 0.5 mL culture medium overnight before mounting on myograph system (Model 700MO; DMTA/S, Aarhus, Denmark) where smooth muscle contractions were evaluated after 24 h culturing with or without monensin (1 or 10 µM) present.

### Tissue organ bath and myography

On experimental days, guinea pig trachea rings or human bronchi sections were mounted in 5 mL-organ bath/myograph chambers with Krebs buffer, which were kept at 37 °C and bubbled with carbogen (5% CO_2_ in 95% O_2_) to keep pH at 7.4. Smooth muscle force was recorded, amplified, and displayed in LabChart software (Version 7.3.8, ADInstruments, New South Wales, Australia). One hour before the pharmacological tests, a gradual increased tension to 30 mN (for guinea pigs) or 1.5 mN (for human bronchi) was applied step-wisely with three washes in between.

To investigate the direct effect of monensin, 1 or 10 µM monensin were added into the baths/chambers with guinea pig tracheal segments and human bronchi for two hours with or without pretreatment with histamine H_1_ receptor antagonist (mepyramine; 1 µM, pretreated for 30 min). The contractions were normalized to the maximal responses induced by histamine (10 nM–1 mM, in guinea pig trachea) and potassium chloride (KCl, 60 mM, in human bronchi).

To study the monensin effect on tissue viability and on histamine responses, segments after culturing for 24–72 h (guinea pig trachea) and 24 h (human bronchi) were exposed to carbachol (1 nM to 0.1 mM) and histamine (10 nM–1 mM). The maximal contractions were referred to as the constriction at the highest concentration of carbachol. For defining the tissue viability, the effect was measured in absolute force (mN).

### Guinea pig in vivo model

Two weeks after HDM sensitization (100 µg/100 mg HDM protein/aluminum hydroxide, *i.p.*), awake guinea pigs were challenged intranasally with 25 µg HDM in 100 µL PBS, once a week for either three or five consecutive weeks. Control animals received 100 mg aluminum hydroxide for sensitization (*i.p.)* and 100 µL PBS for each challenge (*i.n.*). Twenty-four hours before each challenge, animals received 0.5 mg/mL monensin in 200 µL 12.5% ethanol/PBS. Vehicle (12.5% ethanol/PBS) or monensin were administered in control animals. Animal weights were documented before each challenge. One day after the last challenge, animals were anesthetized with *i.p.* injection of 40 mg/kg Ketamine hydrochloride (Ketaminol® Vet., Intervet, Stockholm, Sweden) and 0.5 mg/kg Medetomidine hydrochloride (Cepetor®Vet., VETMEDIC, Stockholm, Sweden). Add-on anesthetics were injected when needed. Animals were canulated and ventilated using the flexiVent system^18^ (flexiVent FX4, SCIREQ Inc, Montreal, Qc, Canada), where airway responses to methacholine (0.03125 mg/mL to 0.25 mg/mL) were assessed. Nebulizer settings: aerosol output rate: 0.40 mL/min, delivery ratio: 54.6%, nebulizer duty cycle: 50%, nebulizing time: 10 s. Naïve animals were used as controls. After airway responsiveness study, animals were euthanized by an overdose of ketamine and medetomidine followed by exsanguination through cardiac puncture.

### Histology

After the pharmacological experiments, guinea pig tracheal segments were fixed in Carnoy solution (60% ethanol, 30% chloroform and 10% acetic acid) for histological analyses. After in vivo experiments, right and left caudal lung lobes were harvested and fixed in Carnoy solution (for Astra blue staining) and 10% neutral buffered formalin (for Hematoxylin–Eosin staining) for further histological processes. Tissues were dehydrated, embedded in paraffin and sectioned into 5 µm slides for staining. To quantify MCs, guinea pig tracheal segments and right caudal lung sections were stained with Astra blue for detection of heparin in MC granule^20^. The staining intensity was scored (1: pale, 2: medium, 3: strong). Astra blue-Hematoxylin staining was used to assess the MC nucleus to identify the death of MCs. Airway inflammation analysis was done on slides stained with Hematoxylin–Eosin (Histolab, Göteborg, Sweden). Pictures of stained slides were taken using Zeiss AxioScan.Z1 Slide Scanner (ZEISS, Ostfildern, Germany) under × 20 magnification. Tracheal rings or all the intact airways found in the stained slides were analyzed. Mast cells were counted manually. The infiltrated inflammatory area and bronchial diameter were calculated using ZEN software (Version 3.3, blue edition, ZEISS, Ostfildern, Germany). All the quantifications were performed blindly and normalized to the diameter of the airways.

### Chemicals and suppliers

Krebs–Henseleit buffer, penicillin, histamine, indomethacin, acetylcholine, potassium chloride, chloroform, acetic acid, mepyramine, monensin, anti-human IgE mAb (product number: I6510) and Astra blue were purchased from Sigma-Aldrich (St. Louis, MO, USA).

### Calculations and statistics

Data presented as mean ± standard error of the mean (SEM). For statistical evaluation, One-way or two-way ANOVA followed by Tukey's multiple comparison tests were performed using GraphPad Prism (version 8.0.1(244), San Diego, California, USA). *p* < 0.05 was considered statistically significant.

### Ethical approval and consent of participation

All experiments related to guinea pigs and human bronchi were approved by Stockholm ethics committee (permit number: 10973-2019 and 2018/1819-31/1). All experiments were performed in accordance with relevant guidelines and regulations. Informed consent of participation from patients were obtained. The study is reported in accordance with ARRIVE guidelines.

## Results

### Monensin exposure reduced MC granule staining intensity and MC number in guinea pig trachea by causing cell death

**Figure 1:**
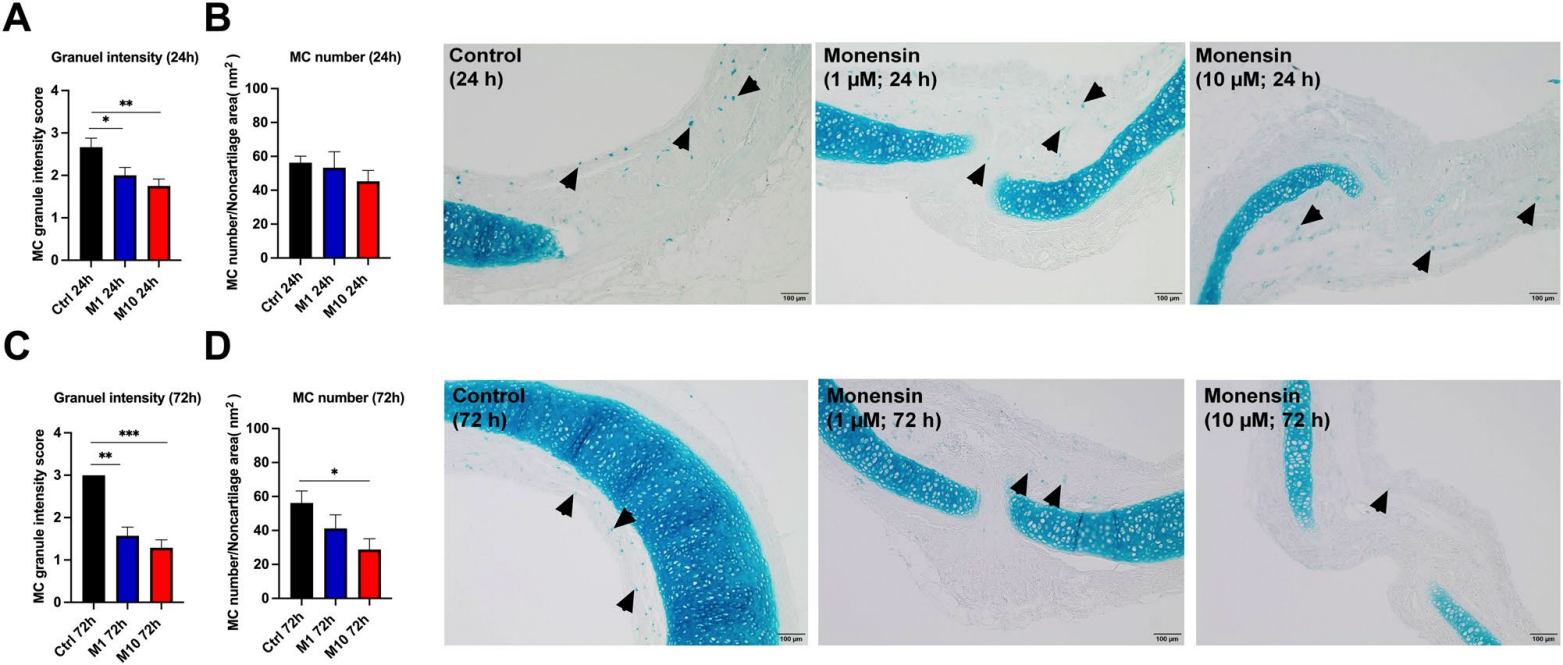
Astra blue staining of guinea pig trachea after 24 and 72 h exposure to monensin. The staining intensity score of mast cells (1: pale, 2: medium, 3: strong) after (**A**) 24 h and (**C**)72 h culture with 1 µM (M1) or 10 µM (M10) monensin, or vehicle (1% ethanol, Ctrl). The mast cell numbers after culturing for (**B**) 24 h and (**D**) 72 h. Representative pictures were listed on the right (n = 6–8 per group). Arrows denote mast cells. * *p* < 0.05, ** *p* < 0.01, and *** *p* < 0.001.

To evaluate the effect of monensin exposure on MCs, tracheal segments cultured with monensin for 24 and 72 h were stained with Astra blue. Notably, the staining intensity of MC granules were found reduced both after 24 h (Fig. 1A) and 72 h monensin exposure with either 1 or 10 µM (Fig. 1C). Mast cell numbers, counted as Astra blue-positive cells, was not significantly changed after 24 h exposure to either 1 or 10 µM monensin (Fig. 1B). In contrast, the quantity of MCs decreased after 72 h monensin treatment, especially at 10 µM (Fig. 1D).

**Figure 2:**
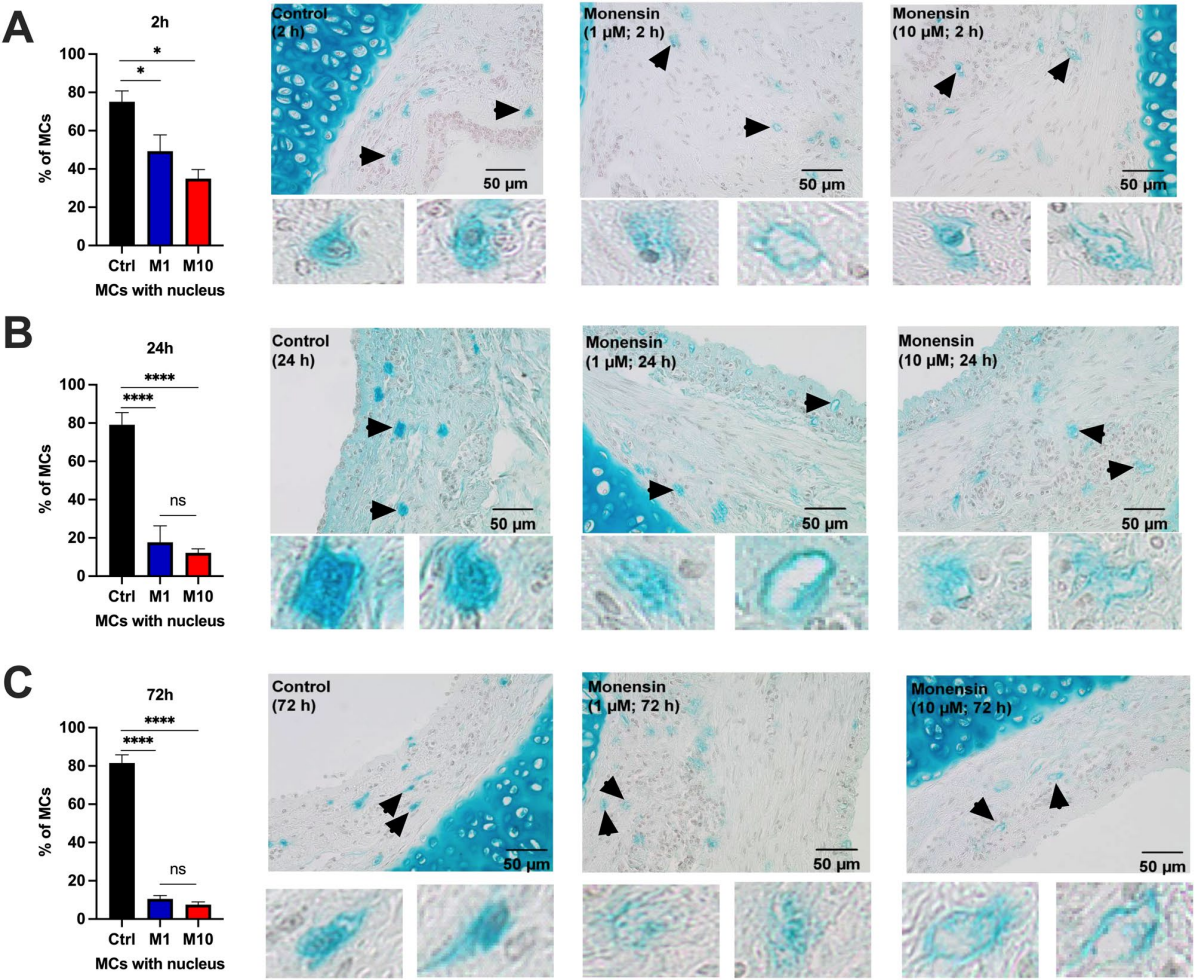
Astra blue-Hematoxylin staining of guinea pig trachea after 2 to 72 h exposure to monensin. Percentage of mast cells that have detectable nuclei after (**A**) 2 h, (**B**) 24 h and (**C**)72 h exposure to 1 µM (M1) or 10 µM (M10) monensin, or vehicle (1% ethanol; Ctrl) with representative pictures on the right (n = 3–8 per group). Arrows denote the magnified mast cells. *p < 0.05 and **** *p* < 0.0001.

To examine whether monensin induce MC death, the guinea pig tracheal segments were stained with Astra blue/Hematoxylin to visualize MC nuclei, after 2–72 h exposure to monensin. As seen in Fig. 2A, 75 ± 6% of the MCs in control segments demonstrated intact nuclei. However, the percentage of MCs with intact nuclei decreased to 49 ± 9% and 35 ± 5% in tissues exposed to 1 µM and 10 µM monensin after 2 h exposure. A marked reduction of intact nuclei was observed, being decreased from 79 ± 6% in the control segments to 18 ± 9% and 12 ± 2% after 24 h exposure to both 1 µM and 10 µM monensin (Fig. 2B). Similar effect was seen after 72 h exposure to monensin at 1 or 10 µM, with the percentage of MCs having intact nuclei being decreased from 82 ± 4 to 11 ± 2% and 8 ± 2% (Fig. 2C).

### Monensin caused direct contraction of guinea pig trachea and human bronchi by histamine release

**Figure 3:**
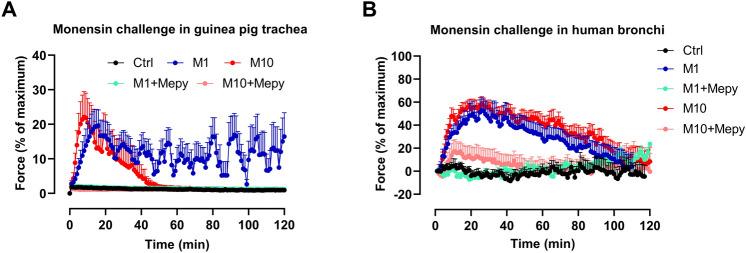
Responses of guinea pig trachea and human bronchi to monensin. (**A**) Smooth muscle contraction of tracheal segments and (**B**) human bronchi to 1 µM (M1) or 10 µM (M10) monensin with or without the presence of mepyramine (1 µM; Mepy) (n = 5–6 per group).

To study if monensin caused a release of contractile agents, segments were exposed directly to monensin and the responses were evaluated in tissue organ bath. It showed that monensin induced contraction of guinea pig tracheal rings to 20 ± 5% (at 1 µM) and 22 ± 7% (at 10 µM) of maximum (Fig. 3A). The contractile response to 10 µM monensin returned to the baseline after 45 min, whereas the contraction to 1 µM monensin was maintained for two hours. The monensin-induced contraction was totally abrogated by pretreatment with 1 µM of the histamine H_1_ receptor antagonist mepyramine.

To examine whether the effect of monensin on guinea pig trachea could be translated into humans, human small airways were isolated and exposed to 1 or 10 µM monensin. Similar to guinea pig trachea, both monensin concentrations induced direct constriction of human bronchi to 53 ± 11% (1 µM) and 59 ± 6% (10 µM) of maximum, which lasted for 2 h. These responses were prevented by pretreatment with 1 µM mepyramine (Fig. 3B).

### Monensin exposure at a low concentration did not affect tissue viability of guinea pig trachea and human bronchi

**Figure 4:**
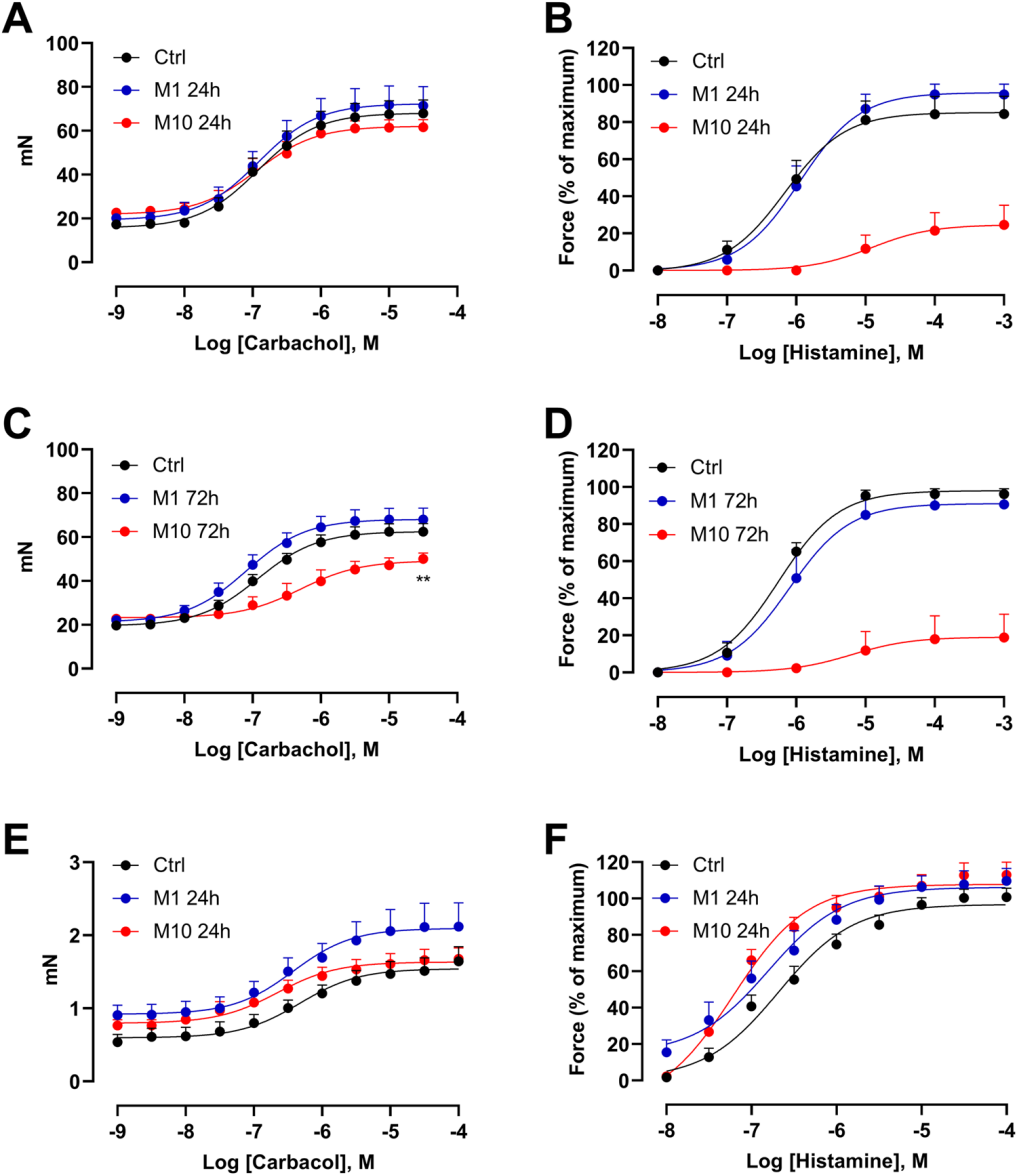
Responses of guinea pig and human airways to carbachol and histamine after monensin exposure. Guinea pig tracheal responses to carbachol after (**A**) 24 h and (**C**) 72 h, and histamine after (**B**) 24 h and (**D**) 72 h exposure to 1 µM (M1) or 10 µM (M10) monensin, or vehicle (1% ethanol; Ctrl). The responses of human bronchi to (E) carbachol and (**F**) histamine after 24 h incubation (n = 6 to 14 per group). ** *p* < 0.01.

To evaluate the monensin effect on tissue viability, the absolute force (mN) to the muscarinic receptor agonist carbachol was measured. In guinea pig trachea, treatment with either 1 or 10 µM monensin had no significant impact on carbachol curves after 24 h culture (Fig. 4A and C). However, the high dose of monensin (10 µM) reduced the effect on the smooth muscle contractile capability to carbachol after 72 h exposure, with pEC_50_ significantly decreased from 6.9 to 6.2 and Emax decreased from 62 ± 4 to 50 ± 3 mN, albeit no such reductions were observed at 1 µM monensin treatment (Fig. 4A and C).

As histamine release was found by monensin, to test whether this release affect the histamine H_1_ receptors or not, histamine with increasing concentrations were given cumulatively to the tissues after monensin exposures. Exposure to 1 µM monensin, either for 24 h or 72 h, had no significant influence on histamine responses (Fig. 4B and D). In contrast, segments exposed to 10 µM monensin displayed both a delayed and reduced reaction to histamine, with a shift of pEC_50_ from 6.1 and 6.3 in the controls to 5.1 and 5.4 together with a decrease of maximal contraction (Emax) from 84 ± 9 and 96 ± 2% to 25 ± 11 and 19 ± 13% after 24 h and 72 h monensin exposure (Fig. 4B and D).

Tissue viability of human bronchi, reflected as responses to carbachol, was not diminished after exposure to monensin (Fig. 4E). As for the histamine response, instead of causing desensitization of the histamine H_1_ receptor, exposure to 10 µM monensin significantly potentiated the histamine response, with a change of pEC_50_ from 6.8 in the control group to 7.2 in the treated group (Fig. 4F).

### Monensin exposure blocked HDM- and anti-IgE induced bronchoconstriction

**Figure 5:**
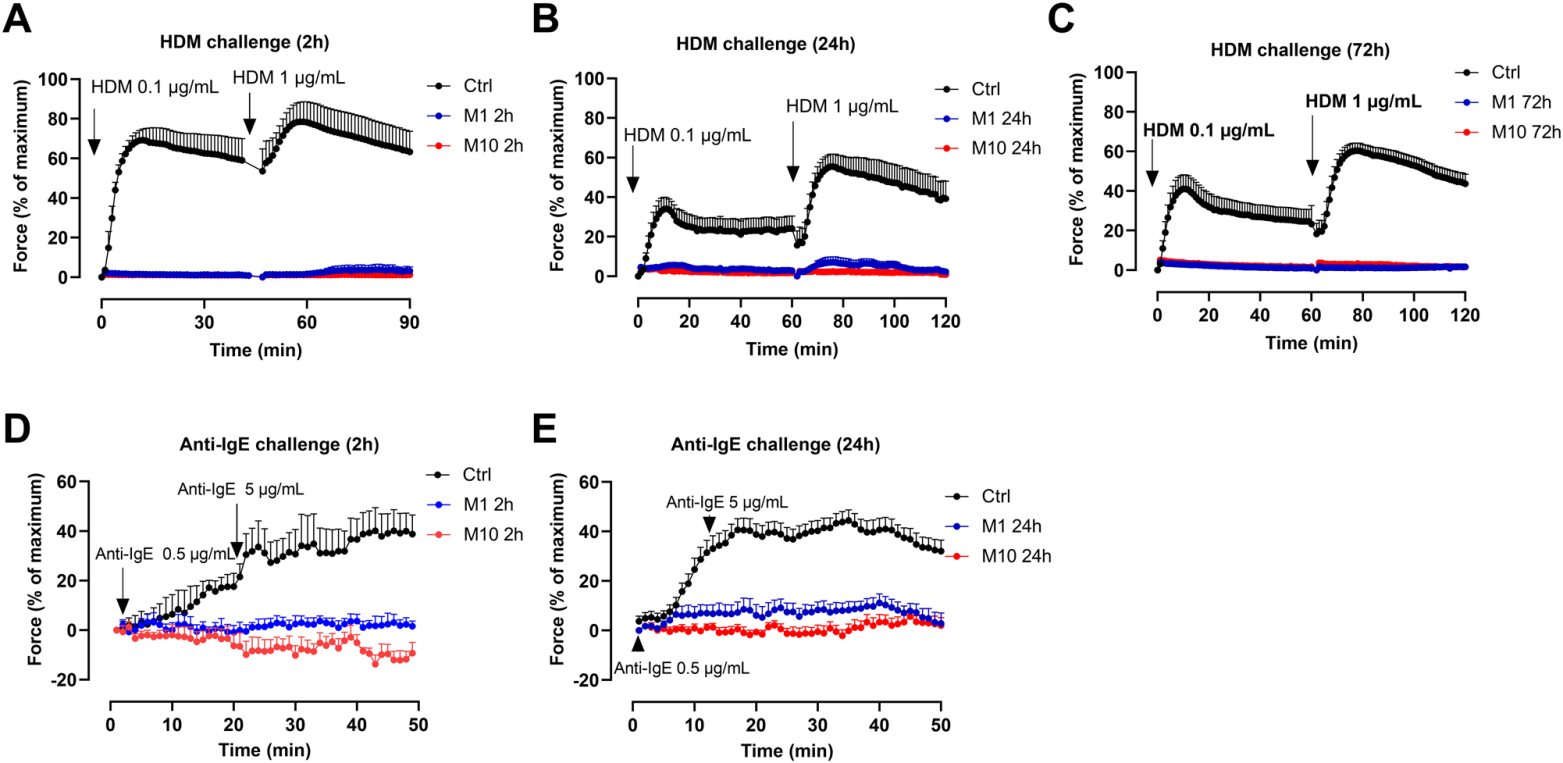
Responses of guinea pig trachea to HDM and human bronchi to anti-IgE after monensin exposure. The responses of guinea pig tracheal segments to HDM after culturing with 1 µM (M1) or 10 µM (M10) monensin, or vehicle (1% ethanol; Ctrl) for (**A**) 2 h, (**B**) 24 h and (**C**) 72 h (n = 3–8 per group). The responses of human bronchi to anti-IgE after (**D**) 2 h and (**E**) 24 h incubation (n = 5–13 per group).

To investigate whether monensin interferes with MC function, HDM challenges (0.1 µg/mL and 1 µg/mL) were performed in guinea pig tracheal segments after 2–72 h monensin treatment. In control segments (for the 2 h exposure), HDM (0.1 µg/mL) induced a strong smooth muscle contraction, reaching 69 ± 6% of the maximum, which was further increased to 79 ± 10% when, additionally, 1 µg/mL monensin was added. No contraction could be detected in the segments pre-exposed to either 1 or 10 µM of monensin for 2 h (Fig. 5A). The contractions induced by HDM (0.1 µg/mL and 1 µg/mL) in the controls reached 34 ± 6% and 55 ± 6% (after 24 h culture) or 41 ± 7% and 60 ± 4% of maximum (after 72 h culture), respectively. However, the antigen-induced contractions were completely abolished after incubation with monensin (1 or 10 µM) for both 24 h and 72 h (Fig. 5B and C).

To study the effect of monensin on MC-related bronchoconstriction in human airways, anti-IgE antibodies (anti-IgE), which bind to IgE at the high affinity IgE receptor on MCs and thereby cause MC activation, were used^11^. As seen in Fig. 5D, 0.5 µg/mL anti-IgE evoked a smooth muscle contraction in control segments (22 ± 5% of maximum) which was further enhanced by 5 µg/mL anti-IgE (40 ± 10%). In contrast, 2 h pre-treatment with either 1 or 10 µM monensin completely prevented the anti-IgE induced constriction (Fig. 5D). Similarly, no bronchoconstriction was detected in tissues treated with either 1 µM or 10 µM monensin for 24 h (Fig. 5E).

### Repeated intranasal dosages of monensin reduced AHR, airway inflammation and mast cell hyperplasia in a guinea pig asthma model

**Figure 6:**
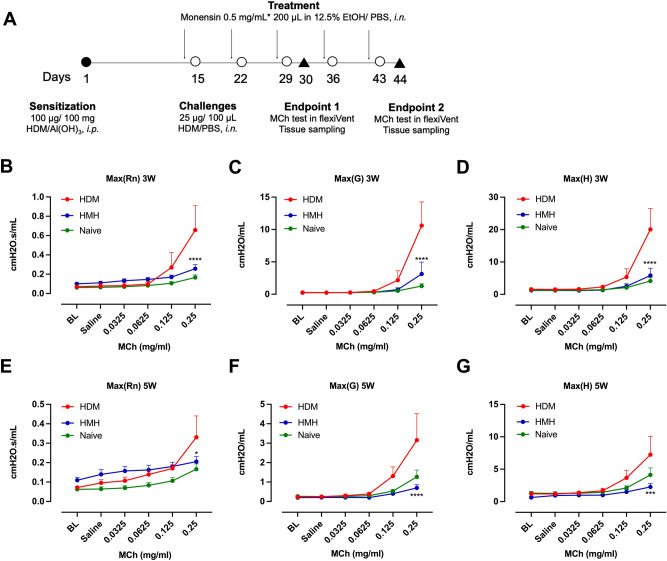
Effect of monensin on airway hyperresponsiveness. (**A**) Schematic diagram of the guinea pig asthma model. Animals were sensitized with a single *i.p.* injection of HDM + adjuvant followed by intranasal HDM challenges for three or five weeks. Monensin (0.5 mg/mL in 200 µL 12.5%EtOH/PBS) was given 24 h before each challenge. Airway responsiveness to methacholine (MCh) was assessed in a flexiVent system one day after (**B–D**) the third or (**E–G**) the fifth challenge. Differences of resistance in conducting airways (*Rn*; **B** and **E**), tissue damping (*G*; **C** and **F**) and tissue elastance (*H*; **D** and **F**) were compared between the animals exposed to HDM with or without monensin treatment (n = 4–7 per group). Control: animals exposed to PBS, received vehicle (12.5% EtOH) or monensin as pretreatment. HDM: animals exposed to HDM and received vehicle as pretreatment. HMH: animals exposed to HDM and received monensin as pretreatment *p < 0.05, ***p < 0.001, ****p < 0.0001.

To investigate the effect of monensin in vivo, a guinea pig asthma model based on HDM exposures was established (Fig. 6A). Compared to the naïve animals, the HDM sensitized animals demonstrated enhanced responses to methacholine after three weeks challenges in the Newtonian resistance (reflecting resistance in conducting airways; *Rn*; Fig. 6B), tissue damping (reflecting resistance in peripheral lung; *G*; Fig. 6C) and tissue elastance (peripheral elastance; *H*; Fig. 6D). However, all the increases were found reduced after pretreatment with monensin (Fig. 6B–D). The exaggerated methacholine responses could also be found after challenges with HDM for five weeks. These increases were also be dampened by monensin (Fig. 6E–G).

**Figure 7:**
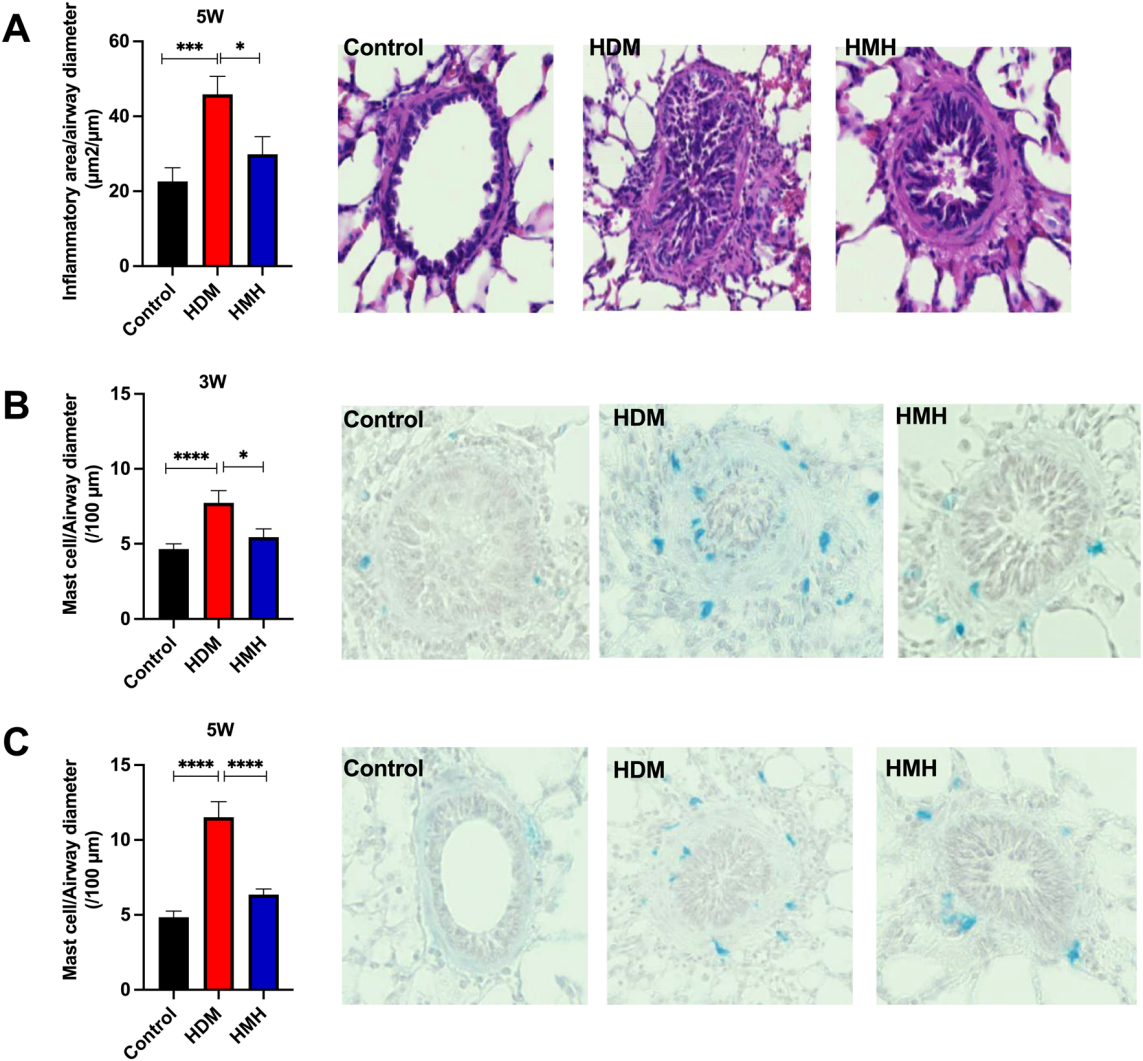
Inflammation and mast cells in airways of guinea pigs. (**A**) Inflammatory areas were compared in Hematoxylin–Eosin-stained slides. Mast cell number was counted in Astra blue stained sections after (**B**) three weeks and (**C**) five weeks challenges. Control: animals exposed to PBS, received vehicle (12.5% EtOH/PBS) or monensin as pretreatment. HDM: animals exposed to HDM and received vehicle as pretreatment. HMH: animals exposed to HDM and received monensin as pretreatment (n = 6–7 per group). *p < 0.05 and *** *p* < 0.001, ****p < 0.0001.

The effect of monensin on airway inflammation was examined in the lung sections stained by Hematoxylin–Eosin from animals received HDM challenges for five weeks. As shown in Fig. 7A, a significant increase of inflammatory area was found in HDM exposed animals. This increase was alleviated by monensin.

To quantify MCs, lung sections were stained with Astra blue/Hematoxylin. After three weeks HDM challenges, an increase of MCs was found (Fig. 7B). This increase remained after five weeks challenges (Fig. 7C). However, the increase of MCs could be reduced by monensin (Fig. 7B and C) to the same level as in controls.

**Figure 8:**
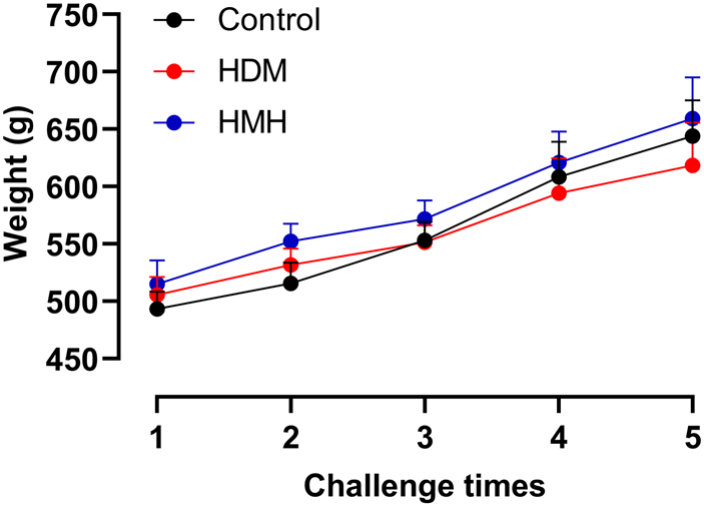
Weight curves of guinea pigs. The weights of animals from the first to the fifth challenge. Control: animals exposed to PBS, received vehicle (12.5% EtOH/PBS) or monensin as pretreatment. HDM: animals exposed to HDM and received vehicle as pretreatment. HMH: animals exposed to HDM and received monensin as pretreatment (n = 6–7 in each group).

Animals tolerated monensin well, with no abnormal behaviors, appearances or significant weight differences compared to control animals (Fig. 8).

## Discussion

This study demonstrated that exposure to monensin in guinea pig trachea reduced MC staining intensity and MC numbers by causing cell death. Direct application of monenin to guinea pig and human isolated airways caused contraction through histamine H_1_ receptors. Exposure to monensin for 2–72 h, completely inhibited the antigen-induced bronchoconstriction in isolated trachea from sensitized guinea pigs. In agreement with these findings, repeated intranasal exposures of monensin reduced AHR, airway inflammation and MC hyperplasia in an in vivo guinea pig asthma model.

Monensin exposure to guinea pig trachea caused an increased proportion of Astra blue-positive cells without intact nuclei suggesting that this drug causes MC death. This is in line with our previous study in which monensin was shown to induce MC death including both apoptosis and necrosis^16^. The proportion of apoptotic and necrotic cells differs depending on MC types used. However, the MC death was predominated by apoptosis^16^. As monensin act as a Na^+^–H^+^ exchanger which changes the granule and cytosolic pH and causing granule permeabilization^16^, the apoptosis might be explained by escaped granule proteases causing induction of pro-apoptotic compounds^12^. However, which type of MC death in guinea pig airways need to be further investigated.

The blockade of monensin-induced bronchoconstriction by histamine H_1_ receptor antagonist in both guinea pig and human airways, indicates that monensin causes histamine release from MCs as monensin itself could not activate histamine receptors (Supplementary Fig. 1). In agreement with this notion, a previous study showed that monensin could induce a dose- and time-dependent histamine release from rat peritoneal MCs^21^. However, apoptotic cell death is typically characterized by the chromatin condensation, DNA fragmentation and formation of apoptotic bodies, but maintained cell integrity without losing cell contents^22^. In contrast, necrosis causes cell membrane damage, karyolysis and loss of membrane integrity, leading to the release of cell contents^23^. Hence, the observed release of histamine suggests that monensin in these settings more likely is due to necrosis of MCs. A possible reason for the difference between the two concentration induced responses is that the higher concentration caused a more rapid release of histamine and it caused a quicker death of MCs, whereas the low concentration induced cell death is slower and therefore the release of histamine is more consistent.

Exposure to monensin (for 2, 24 or 72 h) caused strong inhibition of HDM- and anti-IgE induced bronchoconstriction in guinea pig and human airways. Previous studies have shown that both regimens represent antigen-induced activations of MCs, result in increased release of the MC mediators such as histamine, CysLTs and PGD_2_ from guinea pig trachea^17^ and human MCs^24,25^. It is also known that combined blockade of these MC mediators or their receptors is required to dampen the stimulus-induced smooth muscle contraction^11,17^. Hence, the total blockade of antigen-induced bronchoconstriction of guinea pig trachea and human bronchi suggests that monensin influences these mediators simultaneously, either directly or indirectly. Clearly, this effect is compatible with a scenario in which monensin causes death of airway MCs^16^.

Only 10 µM of monensin exposure for 72 h reduced the responses to carbachol, indicating that monensin at high doses and for prolonged exposure time can cause malfunction of other cells in addition to MCs. The reduced tracheal responses to histamine, together with maintained responses to carbachol after 24 h exposure to 10 µM monensin, suggests a homologous desensitization of the histamine H_1_ receptor, in agreement with a previous study revealing that pre-exposure to histamine could desensitize histamine H_1_ receptors but not muscarinic receptors in guinea pig tissues^26^. In contrast, monensin potentiated the histamine response in human bronchi. This potentiation effect might be due to the release of preformed MC cytokines that are capable of inducing airway hyperreactivity, such as IL-4^27,28^.

In the in vivo study, guinea pigs developed AHR, airway inflammation and MC hyperplasia after repeated HDM exposures. All these were inhibited in animals which were given monensin before each challenge. It is known that HDM exposures can cause MC hyperplasia both in humans^29^ and in animal models^18,30^, which may be due to activation of MCs or other cells, which release chemoattractants that recruit MC progenitors from the circulation^31^. In the present study, repeated exposures to monensin caused a decline in MC numbers to the same level as in controls. However, we cannot at present be certain as to whether this is due to inhibited MC recruitment or due to cell death of resident MCs. As the increase of MCs correlates with AHR^32,33^ and inflammation in asthma^34,35^, it is possible that the reduction of AHR and inflammation in the present study was caused by the decrease of MCs.

In the in vivo study, no abnormal behaviors and weight changes were found in guinea pigs that received monensin treatment. However, monensin has been considered to be a toxic compound with narrow treatment window for horses^36^, cattle^37^ and poultry^38^, causing reduced growth rate in animals receiving high doses when given systemically. Thus, it is possible that the intranasal dose of monensin in this study, which effectively reduced AHR, inflammation and MC hyperplasia in the lung, did not reach toxic systemic levels as intranasal instillation reduces the systemic absorption.

## Conclusion

Our findings show that monensin is an effective tool to reduce MC numbers both in vitro and in vivo. With due consideration of dosing and timing, the study documents that monensin treatment provides a new strategy in experimental asthma and allergy research to elucidate the role of MCs in allergic and non-allergic diseases. Finally, the study highlights that an expansion of MC populations is fundamental for the development of AHR and airway inflammation in the used models which are highly relevant for reactions seen in allergic asthma.

## Supplementary Information

Supplementary Information.

## Data Availability

The associated data in this study are not publicly available due to patenting but will be available upon reasonable request to the corresponding authors.
