# Supplementary material for: Monensin inhibits mast cell mediated airway contractions in human and guinea pig asthma models
Source: Sci Rep. 2022 Nov 7;12:18924. doi: 10.1038/s41598-022-23486-1 (PMC9640546; doi:10.1038/s41598-022-23486-1)
Supplement: Supplementary file 1 — Supplementary Information. [file 41598_2022_23486_MOESM1_ESM.pdf]

Monensin inhibits mast cell mediated airway contractions in human and guinea pig asthma models

Jielu Liu, Mu Nie, Caijuan Dong, Jesper Säfholm, Gunnar Pejler, Gunnar Nilsson, and Mikael Adner

Supplementary Figure:

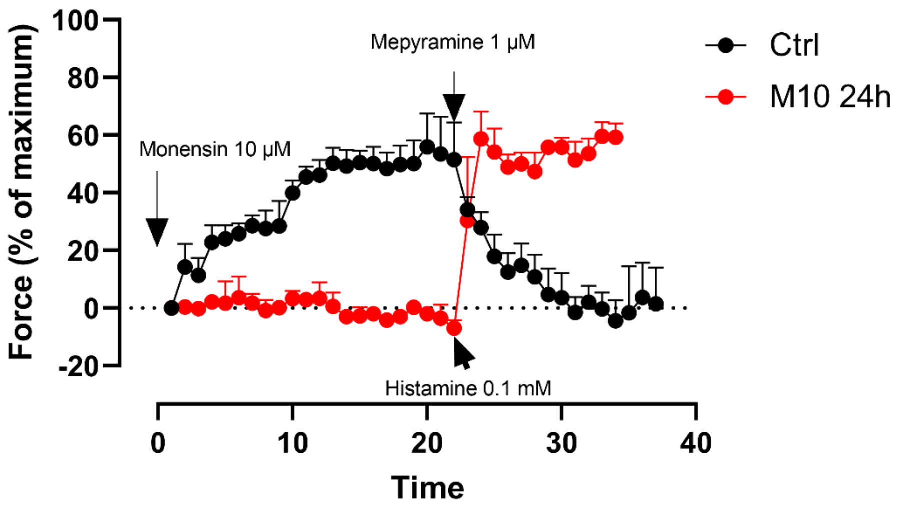

Supplementary Fig 1: Responses of human bronchi to monensin. Segments were cultured with monensin 10 µM (M10) or vehicle (1% ethanol; Ctrl) for 24h before exposing to monensin (10 µM) again in myograph. In Ctrl segments, mepyramine (1 µM) was added at the maximal contraction induced by monensin meanwhile histamine (0.1 mM) was added in the monensin pre-treated segments (n=2 to 3 per group).
